# Influence of various chilling methods on the sustainable beef production based on high voltage electrical stimulation

**DOI:** 10.1371/journal.pone.0240639

**Published:** 2021-11-03

**Authors:** Joanna Katarzyna Banach, Ryszard Żywica, Paulius Matusevičius

**Affiliations:** 1 Institute of Management and Quality, Faculty of Economics, University of Warmia and Mazury in Olsztyn, Olsztyn, Poland; 2 Faculty of Animal Sciences, Veterinary Academy, Lithuanian University of Health Sciences, Kaunas, Lithuania; University of Queensland, AUSTRALIA

## Abstract

Among the challenges of sustainable management of meat production, the key issue is to improve the energy efficiency of production processes, which will consequently affect the reduction of greenhouse gas emissions. Such effects are achieved by combining various chilling systems with electrical stimulation that determines the quality of meat at the slaughter stage. The novelties of the research undertaken included determining the impact of various variants of meat production (chilling method: slow, fast, accelerated + HVES/NES) on changes in the basic (industrial) quality indicators (pH and temperature) of beef produced from Polish Holstein-Friesian breed cattle, and then indicating the optimal variant for energy-efficient (sustainable) beef production. The HVES and the fast chilling method yielded positive economic (meat weight loss), technological (high quality, hot-boning), energetic (lower electricity consumption), and organizational effects (reduced chilling and storage surfaces and expenditures for staff wages) compared to the slow and accelerated methods. Reaching the desired final temperature with an increased amount of chilled meat enables obtaining a few-fold decrease in the specific energy consumption and a higher energy efficiency of the process. This allows recommending the above actions to be undertaken by entrepreneurs in the pursuit of sustainable meat production.

## Introduction

One of the strategies of sustainable production in the agri-food sector, apart from striving to ensure high quality and safety of raw materials, envisages also effective management of energy and the environment throughout the food chain. Pursuant to this strategy, agricultural production is realized through changes in farm management, where energy is used with maximum efficiency (the so-called carbon footprint). Practical tools for the implementation of sustainable beef production at the rearing stage include measures aimed to improve: animal productivity (health and welfare), feed quality, soil fertility, and the efficiency of use of electricity and fertilizers [1]. Proposals for good cattle breeding practices in the Western European region were defined by O’Brien et al. [2]. Among the challenges of sustainable management of production as well as electricity and heat in meat plants, there is a need to, among others, improve production efficiency and reduce energy consumption, including mainly specific energy consumption of production processes, in order to reduce operating costs and mitigate side effects on the natural environment [3, 4]. This is especially important because the meat industry is characterized by a high demand for electricity, as chilling systems consume by far the most of this type of energy [5–7]. In addition, Polish plants with their profitability of meat sales reaching barely 1–2%, deem it necessary to take the above-mentioned measures [8]. However, the investment and modernization activities undertaken at the stage of cattle slaughter and beef carcass/half-carcass chilling need to be adapted to industrial needs, whereas ready-to-adopt technologies should be characterized based on production and energy efficiency indicators of devices/installations and eco-efficiency of plants [9–11].

**Fig 1:**
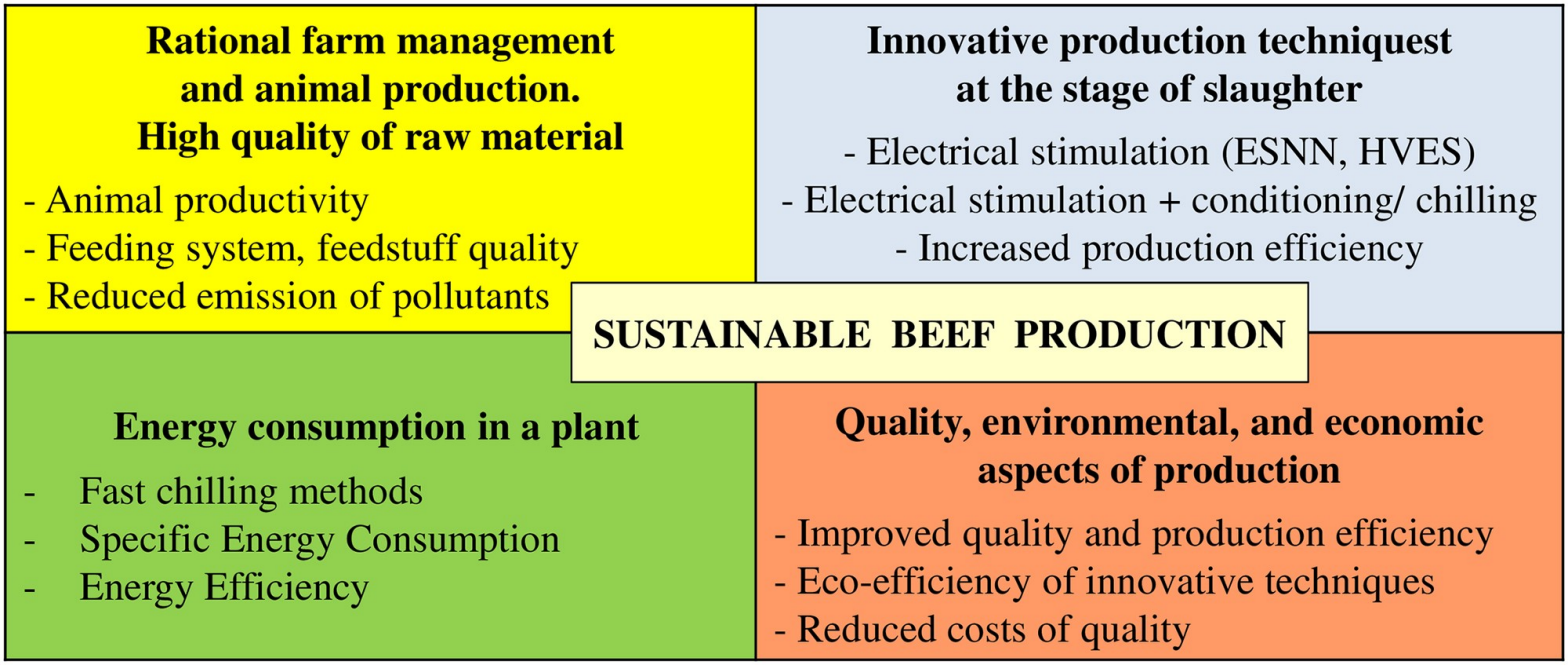
Concept of the sustainable development of beef production.

Today, the development of meat chilling techniques is heading towards systems that do not use extremely low temperatures, and yet allow for low meat weight losses. To improve the energy consumption and economy of beef production, slow methods are replaced by fast and shock methods. In practice, however, too slow or too fast chilling of carcasses may result in their deteriorated quality. Therefore, great caution should be exercised when choosing the right chilling method [12, 13]. An adverse technological phenomenon that can occur during rapid meat chilling is the *cold shortening*. It happens when meat temperature drops below 10°C at a pH value above 6.2, i.e. when the muscles are before or during the *rigor mortis* state. To prevent this phenomenon, various physical methods are deployed to accelerate biochemical changes in meat in the slaughter / pre-chilling period. These include: tender stretching (hanging carcasses by the Achilles tendon / pelvic bone), electrostimulation, or delayed chilling—conditioning [14–17]. Electrical stimulation (ES) is considered an ideal non-invasive solution in this case. However, its implementation in practice depends on the specifics and capabilities of a meat processing plant, including its production efficiency, mechanization of production processes as well as the use of processing power and surfaces [18–20]. Methods used in practice include the low-voltage (ESNN, up to 100V) and the high-voltage one (HVES, from 100 to 3000V) electrical stimulation. The magnitude of their effects on biochemical processes in meat is determined by: animal type, process onset time, and choice of electrical current parameters (frequency, impulse shape, voltage type, etc.). Due to the possibility of exciting muscles through both the nervous system and muscle fiber conductivity, the HVES is considered the most effective method for meat quality improvement [21]. Considering the above, we have developed own concept of the sustainable beef production (Fig 1).

Research trends in the field of combining various red meat cooling systems with available techniques to improve its quality are still investigated [13, 22]. They are also in the focus of interest of entrepreneurs who are obliged to implement the concept of sustainable meat production [11, 23, 24]. The results of our previous investigations [25–28] indicating the beneficial effect of an own-construction device for HVES on the acceleration of post-slaughter changes and on the production of high-quality meat and products made of it (raw and steamed hams; salami-type sausage, beef pastrami) encouraged used to expand the scope of research interests with energy aspects of production. The novelty of the undertaken research lied in determining the quality of meat after HVES in combination with various chilling methods, and then indicating the optimal variant for energy-saving (sustainable) beef production. For this purpose, the aim of the first part of the study was to examine the influence of various variants of meat production (chilling method: slow, accelerated, fast, + ES/NES) on changes in industrial quality parameters (pH, temperature) of beef obtained from Polish Holstein-Friesian breed cattle, whereas that of the second part was to analyze energy consumption and weight loss of meat in the most advantageous production variant (fast chilling + ES), taking into account the varied amount of raw material chilled in the chamber.

## Material and methods

The research material consisted of carcasses/half-carcasses of meat cattle (heifers, bulls—age around 18 months, cows—age around 10 years) of the Polish Holstein-Friesian Black and White breed. The hot carcass standard weight was approx. 240 kg (min.178.9^±^27.7kg; max. 298.5^±^41.5 kg). Stunning and post-stunning procedures were executed under industrial conditions following provisions of the Council Regulation (EC) No 1099/2009 of 24 September 2009 on the protection of animals at the time of killing. The study was carried out in a medium-sized meat processing plant with a capacity of the slaughter line reaching 100 carcasses per shift. The slaughter was controlled by the State Veterinary Inspector to keep required conditions concerning animal welfare and especially to minimize suffering. The slaughter was done by qualified persons using a pneumatic cattle gun.

### High-voltage electrical stimulation (HVES)

Approx. 40 min, after stunning, carcasses were treated with a high-voltage electrical stimulation (HVES) device designed by Żywica, & Banach [29]. The left half-carcasses were subjected to ES using alternating electrical current with the following parameters: U = 330V, f = 17Hz, rectangular shape of impulses, and pulse-duty factor 0.9 for 120 sec. The right half-carcasses served as the control non-electrostimulated sample (NES). Afterwards, the half-carcasses were washed with water having a temperature of ca. 10°C, weighed, and transferred to chilling chambers. This comparative system (ES/NES) was used to determine the changes in the pH values of meat of heifers, bulls, and cows since stunning. In the last stage of the study addressing the energy balance of the fast chilling method, ES was applied to whole carcasses.

### Chilling methods

The beef half-carcasses were chilled with the slow, accelerated, and fast two-phase methods (60 animals in each method—I stage), under the following conditions:

1. ***Slow method (delayed chilling)***: After leaving the slaughter line, the half-carcasses were transferred to a chilling chamber with air temp. of ca. +10°C, where they were kept for ca. 6h until the chamber had been filled up. Afterward, cooling aggregators were turned on and the half-carcasses were chilled with air having a temperature of ca. 2°C and humidity of ca. 90% for 24h.
2. ***Accelerated method***: After transportation to the chilling chamber, the half-carcasses were chilled with air having a temperature of ca. 2°C and humidity of ca. 90% for 24h.
3. ***Fast two-phase method***: After leaving the slaughter line, the carcasses ES were transferred to a fast chilling chamber with air temp. of ca. -8°C and humidity of 95%, where they were kept for 2.5h. Afterward, they were transferred to an equalizing (transitory) chamber to equalize temperatures of their outer and inner layers (for 0.5-3h depending on the number of half-carcasses), with the fans switched off. Next, they were dissected into quarter-carcasses and transferred to a chilling tunnel with air temp. of 0–2°C and humidity of ca. 90% for ca. 16-18h.

In all chilling chambers, the half-carcasses were moved in a continuous pendular motion with strong forced convection.

### Temperature measurements

Meat temperature measurements were performed over the chilling time using a TES 1310 TYPE-K spike thermometer (accuracy of ^±^0.1°C), at three different localizations of non-stimulated (NES) half-carcasses: *semimembranosus* muscle, *longissimus dorsi* muscle, and around a shoulder blade, at a depth of ca. 7cm. The measurements were performed 1, 3.5, 8, and 24h after stunning, and their results are presented in Table 1 as mean values ± standard deviation. The number of examined carcasses reached n = 60 in each chilling method. Due to the harsh industrial conditions (low temperatures and very high air humidity) and the need to obtain approval from plant management to enter the chilling chambers, the number of measuring points was reduced to the necessary minimum.

**Table 1:** Temperature changes (mean values ± standard deviation) during NES-beef chilling with the slow (SM), accelerated (AM), and fast (FM) methods.

Experimental group		Chilling methods			Significance		
		SM (n = 60)	AM (n = 60)	FM (n = 60)			
					SM-AM	AM-FM	FM-SM
**Time after stunning [h]**	1	38.8 ^±^ 0.69^a^	38.6 ^±^ 0.57^a^	38.2 ^±^ 1.04^a^	NS	NS	NS
	3.5	35.7 ^±^ 1.15^b^	25.7 ^±^ 3.18^b^	18.1 ^±^ 1.57^b^	**	**	**
	8	25.3 ^±^ 2.22^c^	11.9 ^±^ 2.58^c^	10.3 ^±^ 1.68^c^	**	NS	**
	24	4.8 ^±^ 0.45^d^	3.8 ^±^ 0.79^d^	1.4 ^±^ 0.95^d^	**	**	**

^a-d^—means in columns with different letters differ significantly at *p<0*.*01*;

NES-beef: non-electrostimulated beef.

**—significance level *p<0*.*01*;

NS—non-significant.

### pH measurements

Measurements of pH of stimulated (ES, n = 15) and non-electrostimulated (NES, n = 15) beef chilled with the slow, accelerated, and fast methods were performed 2/3, 2, 6, and 24h after stunning with a pH-meter (CP-411, electrode OSH 12–01, Elmetron, Poland) in three different localizations in *m*. *longissimus thoracis et lumborum* between 7 and 8^th^ rib. The decision to choose *m*. *longissimus* as the representative muscle for pH measurements was driven by the results our previous research [30–32]. Before the measurements made for meat from a given experimental group (ES, NES), the electrode was washed with distiller water and the pH-meter was calibrated.

### Weight losses of meat (WLM)

The effect of the fast chilling method on weight losses of stimulated meat (WLM) was determined based on the difference between the weight of carcasses (n = 390) before (M_i_) and after (Mf) the chilling process (initial—W_i_ and final—W_f_ weight, respectively) and expressed in per cents (Eq 1).


$$\text{WLM}=\left({\text{M}}_{\text{i}}–{\text{M}}_{\text{f}}\right)/{\text{M}}_{\text{i}}\cdot 100\left[\%\right]$$ (1)

### Energy balance, specific energy consumption, and energy efficiency

The heat balance of the chilling chambers used in the fast chilling method was based on the amount of heat necessary to be removed from the fast chilling chamber (area of 84 m^2^) and the chilling tunnel (area of 57 m^2^) during one chilling cycle. The measurements were carried out in the spring season for 8 working days (D1-D8), with varying amounts of carcasses (n = 10 to n = 144) in the chamber.

The total heat (Q) collected from the chilling chamber (Eq 2) and its components were calculated using the following formulas (Eqs 3–6):

$$Q=Q_{1}+Q_{2}+Q_{3}+Q_{4}+Q_{d}\left[\text{kJ}\right]$$ (2)

where; Q_1_—heat removed form products (half-carcasses);

Q_2_—heat transferring to the environment; the so-called transfer heat;

Q_3_—ventilation heat;

Q_4_—engine work heat;

Q_d_—additional losses, in small chambers.

$$Q_{1}=M\;c_{w}(t_{p}-t_{k})\left[\text{kJ}\right]$$ (3)

where: M—weight of chilled half-carcasses [kg]–before transfer to the chilling chamber,

c_w_—specific heat capacity of half-carcasses [2.85 kJ/kg °C],

t_p_—mean initial temperature of half-carcasses [°C]

t_k_—mean final temperature of half-carcasses [°C]

$$Q_{2}=3600\;k\cdot F\left(t_{z}–t_{w}\right)\cdot \tau \left[\text{kJ}\right]$$ (4)

where: k—heat transfer coefficient of a building partition [0.44 W/m^2^ K]

F—size of a building partition [m^2^],

t_z_—mean outer temperature of chamber environment [ºC],

t_w_—mean inner temperature of the chamber [ºC],

**τ**—heat transfer time [h].

The size of a building partition was assumed to be the size of the outer walls and ceiling of the chamber.

$$Q_{3}=n\cdot V\cdot d\left(i_{1}–i_{2}\right)\;\;\left[\text{kJ}\right]$$ (5)

where: n—number of air exchanges in a chamber, for chilling chambers: n = 2 per day

V—cubic capacity of a chamber [m^3^],

d—mean air density in a chamber [kg / m^3^],

i_1_—fresh air enthalpy [kJ / kg],

i_2_—enthalpy of air in a chamber [kJ / kg].

Engine work heat (Q_4_) was assumed to be the electric energy consumed by engines installed in the chamber. It was calculated based on engine power and work time.


$$Q_{d}=0.15\;\left(Q_{1}+Q_{2}+Q_{3}+Q_{4}\right)\left[\text{kJ}\right]$$ (6)

The heat of lighting and the heat of men’s work did not occur in the applied chilling technology. Data regarding: casing losses (Q_2_ –heat penetrating from the environment), total lighting, engine work, losses due to door opening, and fan work were derived from the ‘Operation and Maintenance Documentation’ available at the technical department of the audited plant.

Energy consumption of the production process, meaning the demand for heat energy necessary to accomplish a specified production process, was determined based on measurements of electric energy consumption by chilling devices (compressors, FChCh+ChT). Indicators of the total specific energy consumption (SEC_1_ and SEC_2_) and energy efficiency, i.e., reduction of energy consumption (EE_1_, EE_2_), per number or weight of slaughtered animals, were used to determine the effect of employing innovative solutions (fast chilling method + HVES). They were computed using the following formulas (Eqs 7–10):

$$SEC_{1}=Q/Z_{1}\;\left[\text{kJ}/\text{number}\;\text{of}\;\text{carcasses}\right]$$ (7)

$$SEC_{2}=Q/Z_{2}\;[\text{kJ}/\text{kg}]$$ (8)

$$EE_{1}=\;Z_{1}/Q\;\left[\text{number}\;\text{of}\;\text{carcasses}/\text{kJ}\right]$$ (9)

$$EE_{2}=Z_{2}/Q\;\left[\text{kg}/\text{kJ}\right]$$ (10)

where: **Q**_**d**_—total heat removed from chilling chambers—FChCh + ChT,

**Z**_**1**_—number of carcasses (number of slaughtered animals),

**Z**_**2**_—weight of slaughtered animals [kg].

**SEC**_**1**_; **SEC**_**2**_—Specific Energy Consumption per number / weight (initial) of slaughtered animals [kJ /number of carcasses] / [kJ / kg],

**EE**_**1**_; **EE**_**2**_—Energy Efficiency per number or weight of slaughtered animals.

### Statistical analysis

The statistical analysis of the effects of chilling duration and method and of electrical stimulation on changes in meat temperature and pH was carried out using *Statistica* 13.1 software based on one-way analysis of variance (ANOVA, *p<0*.*01*, *p<0*.*05*).

## Results and discussion

### Effect of the chilling method on changes in temperature of non-electrostimulated (NES) meat

Analyses carried out in the first part of the study were expected to allow understanding the mechanisms of the impact of various chilling methods on the basic meat quality attributes, expressed by changes in the temperature inside the meat and in meat pH, determining the rate of post-stunning changes, as well as quality and safety of beef during further storage or processing.

Results of meat temperature measurements demonstrated that the type of the chilling method (slow, accelerated, fast) had a significant effect on the rate of changes in muscle tissue temperature. Beef chilling with the slow method caused very slow but significant (*p<0*.*01*) changes in its temperature. The storage of beef half-carcasses in a chilling chamber for 2.5h at the elevated temperature (ca. +10°C/6h) contributed to meat temperature decrease by barely ca. 3°C, i.e. to ca. 36°C. In turn, after 8 and 24h of slow chilling, meat temperature dropped to ca. 25 and 5°C, respectively. Compared to the slow chilling method, slightly higher rate and significant (*p<0*.*01*) changes in meat temperature were demonstrated during accelerated chilling. As soon as after 2.5h of chilling, meat temperature decreased by ca. 13°C and reached ca. 26°C. After 8h and 25h after stunning, it reached ca. 12°C and 4°C, respectively (Table 1). The above results corroborate literature data indicating that achieving this rate of changes in meat temperature in the first hours of post-stunning slow and accelerated chilling can pose the risk of microbiological contamination caused by the rapid proliferation of microorganisms on the surface of half-carcasses [21]. In contrast, the rate of changes in meat temperature was consistent with the ‘10/10 rule of thumb’, indicating that meat chilling to a temp. not lower than 10°C within 10 h post-slaughter, at pH below 6.2, allows avoiding the ‘cold shortening’ phenomenon. The effect of counteracting this adverse phenomenon can be intensified by delayed/slow chilling, which involves keeping the half-carcasses outside the chilling room for some time [33] or under either elevated temperatures, i.e., 10–16°C / 3-12h [34–36] or lower temperatures, i.e., 5–6°C / 24h [37].

The chilling of beef half-carcasses with the fast two-phase method had a strong effect on meat temperature decrease compared to the slow and accelerated methods. Keeping the half-carcasses in the chilling chamber for 2.5h caused its temperature to drop from ca. 38°C to ca. 18°C. During further meat keeping in a chilling tunnel, its temperature dropped to ca. 10°C after 8h and to 1.4°C after 24h. The final temperature of meat chilled with the fast two-phase method was lower than of the meat chilled with the slow (ca. 5°C) and accelerated (ca. 4°C) methods. The statistical analysis of the effects of chilling methods on changes in meat temperature also demonstrated significant (*p<0*.*01*) differences between mean meat temperatures recorded after 3.5 and 24h of chilling with SM, AM, and FM as well as between mean meat temperatures measured after 8h of chilling with SM/AM and FM/SM (Table 1).

### Effect of chilling method and electrostimulated on changes in meat pH

The subsequent part of the study aimed to determine the effect of different meat production variants (chilling method slow, fast, accelerated + ES/NES) on changes in the industrial indicators (pH and temperature) of beef quality [32, 38]. This complex approach was expected to allow identifying the optimal variant of meat production, ensure its high quality, and reduce its harmful effect on environment.

Results of pH measurements of the *longissimus dorsi* muscle of ES and NES heifers, cows, and bulls chilled with the slow method showed that ES significantly (*p<0*.*001*) accelerated the post-stunning transformations during 24-h chilling. The highest and significant differences in pH values were obtained after 2 and 6 h of chilling when meat temperature was very high (ca. 37–30°C). The pH_2h_ value of ES heifer, cow, and bull meat ranged from 5.98 to 6.10 and was lower by ca. 0.9 units compared to the pH_2h_ value of control samples. After 6h post-stunning, the pH value of ES meat was still significantly lower (by 0.7–0.8 units, *p< 0*.*001*) compared to the pH values of meat of non-stimulated heifers, bulls, and cows that reached 6.42, 6.54, and 6.63, respectively. The final pH values (pH_24h_) of both ES and NES meat were similar and reached before 5.7; however, the pH_24h_ values of ES and NES meat of heifers and cows differed significantly at *p<0*.*05* (Table 2). The analysis of meat temperature recorded after ca. 3.5h of chilling (Table 1) and pH value of ES meat measured 2h post-stunning allows concluding that the use of HVES enables the hot-boning of meat as soon as 2h after stunning without the fear of its cold shortening during consecutive ripening. Moreover, electrostimulation allows eliminating the phase of meat retention in the chilling chamber (2.5h/ ca.10°C) and increasing production surface [39, 40].

**Table 2:** Changes in the pH value ($\bar{x}$ ± SD) of the *longissimus dorsi* muscle of non-electrostimulated (NES) and electrostimulated (ES) heifers, cows, and bulls chilled with the slow method.

Experimental group		Heifers			Cows			Bulls		
		ES (n = 15)	NES (n = 15)	Significance	ES (n = 15)	NES (n = 15)	Significance	ES (n = 15)	NES (n = 15)	Significance
**Time after stunning [h]**	2/3	6.95 ^±^ 0.10^a^	7.02 ^±^ 0.09^a^	NS	6.98 ^±^ 0.11^a^	7.00 ^±^ 0.06^a^	NS	6.86 ^±^ 0.11^a^	6.91 ^±^ 0.08^a^	NS
	2	5.98 ^±^ 0.05^b^	6.61 ^±^ 0.05^b^	***	6.10 ^±^ 0.08^b^	6.87 ^±^ 0.04^b^	***	6.03 ^±^ 0.09^b^	6.66 ^±^ 0.02^b^	***
	6	5.75 ^±^ 0.04^c^	6.42 ^±^ 0.03^c^	***	5.79 ^±^ 0.07^c^	6.63 ^±^ 0.02^c^	***	5.82 ^±^ 0.10^c^^C^	6.54 ^±^ 0.05^c^	***
	24	5.64 ^±^ 0.05^d^	5.73 ^±^ 0.06^d^	*	5.69 ^±^ 0.03^d^	5.75 ^±^ 0.05^d^	*	5.70 ^±^ 0.12^D^^d^	5.78 ^±^ 0.03^d^	NS

^**a–d**^—means in the columns with different superscripts are significantly different, P<0.01.

***—significance level P < 0.001;

**—significance level P < 0.01;

*—significance level P < 0.05;

NS—non-significant.

The analysis of pH values of meat chilled with the accelerated method (AM) demonstrated the rate of changes in pH of NES meat over the chilling period had similar values (did not differ significantly) as in the slow method. Therefore, these changes were not presented in Table 2. In turn, the pH values of ES meat of heifers, cows, and bulls were insignificantly higher than in the slow chilling method (SM). The pH values measured in ES meat 2, 6, and 24h post-stunning fitted within the following ranges: pH_2h_ = 6.01–6.06, pH_6h_ = 5.73–5.87, and pH_24h_ = 5.6–5.7. The gender of animals had no significant effect on the final pH values (pH_24h_) of ES meat. However, the pH_24h_ value of bulls was higher and more differentiated (5.73^±^0.17) compared to the pH_24h_ value of heifers (5.60^±^0.05) and cows (5.65^±^0.04)—Table 3. Higher and more differentiated pH values of meat of the stimulated bulls proved that bulls are more susceptible to the pre-slaughter stress than heifers and cows [21]. Negligible differences in the rate of pH changes between ES and control meat indicate that the AM chilling method is more rational and efficient than the SM, from the practical point of view.

**Table 3:** Changes in the pH value ($\bar{x}$ ± SD) of the *longissimus dorsi* muscle of electrostimulated (ES) heifers (H), cows (C), and bulls (B) chilled with the accelerated method*.

Experimental group		Heifers ES (n = 15)	Cows ES (n = 15)	Bulls ES (n = 15)	Significance		
					H-C	C-B	B-H
**Time after stunning [h]**	2/3	7.00 ^±^ 0.07^a^	6.98 ^±^ 0.07^a^	6.89 ^±^ 0.10^a^	NS	NS	NS
	2	6.01 ^±^ 0.04^b^	6.06 ^±^ 0.05^b^	6.03 ^±^ 0.13^b^	NS	NS	NS
	6	5.73 ^±^ 0.05^c^	5.78 ^±^ 0.09^c^	5.87 ^±^ 0.13^c^	NS	NS	NS
	24	5.60 ^±^ 0.05^d^	5.65 ^±^ 0.04^d^	5.73 ^±^ 0.17^c^	NS	NS	NS

^**a–d**^—means in the columns with different superscripts are significantly different, P<0.01;

NS—non-significant

*changes in pH of NES meat over the chilling period had similar values (did not differ significantly) as in the slow method

The use of lower temperatures during the fast chilling of ES meat (-8°C/2.5h, 1–2°C/ca. 16-18h) than in the accelerated and slow methods contributed to the slower post-stunning biochemical changes of meat and, consequently, to pH values higher by ca. 0.3 units at particular measuring points. While the temperature of stimulated meat chilled with the fast method reached 18 and 10°C after 2 and 8h since stunning, its pH_2h_ was at ca. 6.2 and 6.3, and its pH_6h_ was at 5.95 (Table 4). This means that the coupling of HVES treatment performed with the own-construction device and the fast chilling method allows achieving a very rapid temperature drop before the onset of *rigor mortis* and producing high-quality meat without the fear of its cold shortening [41–44]. In the case of NES meat, at pH_2h_ = 6.7–6.8 and pH_6h_ = 6.5–6.6, the biochemical processes are still in progress, and meat is still before the *rigor mortis* state; therefore, its rapid chilling without ES is not recommendable [45].

**Table 4:** Changes in the pH value ($\bar{x}$ ± SD) of the *longissimus dorsi* muscle of non-electrostimulated (NES) and electrostimulated (ES) heifers (H), cows (C), and bulls (B) chilled with the fast method.

Experimental group		Heifers (n = 15)			Cows (n = 15)			Bulls (n = 15)		
		ES	NES	Significance	ES	NES	Significance	ES	NES	Significance
**Time after stunning [h]**	2/3	6.87 ^±^ 0.11^a^	6.91 ^±^ 0.05^a^	NS	7.02 ^±^ 0.06^a^	7.00 ^±^ 0.03^a^	NS	6.92 ^±^ 0.10^a^	6.95 ^±^ 0.09^a^	NS
	2	6.24 ^±^ 0.08^b^	6.67 ^±^ 0.02^b^	***	6.29 ^±^ 0.11^b^	6.80 ^±^ 0.04^b^	***	6.35 ^±^ 0.07^b^	6.71 ^±^ 0.06^b^	**
	6	5.95 ^±^ 0.12^c^	6.50 ^±^ 0.07^c^	***	5.92 ^±^ 0.09^c^	6.65 ^±^ 0.04^c^	***	5.95 ^±^ 0.11^c^	6.53 ^±^ 0.07^c^	***
	24	5.69 ^±^ 0.06^d^	5.78 ^±^ 0.06^d^	*	5.74 ^±^ 0.10^d^	5.72 ^±^ 0.08^d^	NS	5.70 ^±^ 0.05^d^	5.75 ^±^ 0.04^d^	NS

^**a–d**^—means in the columns with different superscripts are significantly different, *p****<****0*.*01*.

***—significance level *p<0*.*001*;

**—significance level *p<0*.*01*;

*—significance level *p<0*.*05*;

NS—non-significant.

Given the results presented above, it has been concluded that the coupled use of HEVS performed using an own-construction device and a fast chilling method is justified considering both the economic and meat quality class established based on pH [32].

### Energy consumption of the meat chilling with the fast method

The rational management of electrical energy in the production process entails not only accomplishing economic effectiveness (increasing savings) but also reducing the energy consumption of technological processes and facilities, which determine the magnitude of effects of technical and man activities on the natural environment [23, 46]. The chilling systems are the largest electricity consumers in the meat industry–they account for 50–93% of the total electric energy consumed in a typical slaughterhouse [5, 47]. Therefore, as part of internal cost control / production savings and preparation for the energy audit [48], entrepreneurs (with individual production specifics) should keep records of the total consumption of energy carriers. In addition, determination of the electric energy consumption of production processes allows entrepreneurs to compare their production expenditures with those of the competition. The extent of implementing the sustainable energy policy in the meat (food) industry is most of expressed by the specific energy consumption–SEC [49–51].

Results of measurements and calculations of the heat balance of the fast chilling chamber (FChCh) and the chilling tunnel (ChT) demonstrated that the heat of penetration, lighting heat, and engine work heat in these rooms had constant values (FChCh = 33.94 kJ/h, ChT = 23.26 kJ/h). The total amount of heat necessary to be removed from the chambers depended on the duration of their work and the amount of raw material being chilled (half-carcasses, quarter-carcasses). In turn, the weight and type of chilled raw material, as well as the final temperature of chilled meat (0–2°C) determined the amount of heat that had to be removed from it [52, 53]. Apart from the mentioned factors, the total amount of heat depended on additional heat losses associated with door opening (10–15%) and heat emitted by ventilation fans (20%)–Table 5.

**Table 5:** Energy balance, Specific Energy Consumption (SEC), and Energy Efficiency (EE) for the fast chilling process, and changes in weight losses of electrostimulated meat during 8 days (D1-D8).

No	Specification		Unit	D1 (n = 10)	D2 (n = 21)	D3 (n = 26)	D4 (n = 42)	D5 (n = 50)	D6 (n = 59)	D7 (n = 68)	D8 (n = 114)
1	**Casing losses (Q**_**2**_ **+Q**_**4**_ **)**	FChCh	kJ/h	33.94							
		ChT		23.26							
2	**Work time**	FChCh	h	3.5	4.5	5.0	6.5	8.0	7.5	7.5	8.5
		ChT		18.0	18.0	17.5	16.0	14.5	15.0	15.0	14.0
3	**Total housing losses** (**1×2)**	FChCh	kJ	118.79	152.73	169.70	220.61	271.52	254.55	254.55	288,49
		TCh		418.68	418.68	407.05	372.16	337.27	348.90	348,9	325.64
4	**Heat removed from half-carcass (Q**_**1**_ **)**	FChCh	kJ	178.90	325.72	347.66	600.64	609.11	616.41	622,73	1009.70
		ChT		61.90	147.56	158.27	264.99	291.23	322.63	386.56	631.84
5	**Heat removed** (**3+4)**	FChCh	kJ	297.69	478.45	517.36	821.25	880.63	870.96	877.28	1298.19
		ChT		480.58	566.24	565.32	637.09	628.50	671.53	735.46	957.48
6	**Open-door losses (Q**_**d**_ **)**	FChCh	%	10							
		ChT		15							
7	**Ventilation heat losses (Q**_**3**_ **)**		%	20							
8	**Total heat removed from**:	FChCh	kJ	391.54	632.99	684.57	1084.05	1174.82	1145.25	1158.00	1717.23
		ChT		660.52	775.42	797.18	879.14	898.48	966.57	1014.83	1331.37
9	**Heat removed per carcass Total heat removed (5+6+7)**	FChCh	kJ/carcass	39.15	30.14	26.33	25.81	23.50	19.41	17.03	15.06
		ChT		60.05	36.40	30.67	20.93	17.97	16.38	14.92	11.68
10	**Electric energy consumed by compressors**		kJ	460.77	683.99	740.52	1009.89	1062.53	1089.14	1160.62	1746.20
11	**SEC**_**1**_		kJ/carcass	46.08	32.57	28.48	24.05	21.25	18.46	17.07	15.32
	**SEC**_**2**_		kJ/kg	0.154	0.123	0.112	0.098	0.108	0.103	0.096	0.056
12	**EE**_**1**_		carcass /kJ	46.08	32.57	28.48	24.05	21.25	18.46	17.07	15.32
	**EE**_**2**_		kg/kJ	0.154	0.123	0.112	0.098	0.108	0.103	0.096	0.056
13	**Initial weight (W**_**i**_ **)**		kg	2985	5569	6616	10331	9866	10553	12072	31437
14	**Final weight (W**_**f**_ **)**		kg	2842	5369	6411	9969	9639	10300	11746	30620
15	**Weight losses of meat (WLM)**		%	4.8	3.1	3.1	3.5	2.3	2.4	2.7	2.6
16	**Average weight loss of meat (n = 390)**		%	2.8							

Q_2_—heat transferring to the environment; the so-called transfer heat; Q_4_—engine work heat; FChCh—fast chilling chamber; ChT—chilling tunnel.

Results of measurements and calculations demonstrated that the total heat needed to be removed from chilling chambers increased along with the increasing number of carcasses intended for chilling (10–114 animals). However, ca. 11-fold increase in the number of chilled carcasses caused over 4-fold increase (391.54–1717.23 kJ) in the fast chilling chamber (FChCh), and a 2-fold increase (660.52–1331.37 J) in the chilling tunnel (ChT) in the amount of heat that had to be removed from these rooms (Table 5). This indicates that the heat load of the chilling chamber (production of the amount of chill and electrical energy consumption) increases along with the increasing load of products placed in it within a day, with the increasing specific heat of the chilled product, and with the increasing difference of temperatures [47]. Keeping the temperature lower than the ambient temperature in cold rooms requires also discharging heat energy from them, which entails higher losses (longer work of compressors) that will generate greater costs of energy consumption. The amount of heat that needs to be removed from chilling chambers is also strongly affected by the ambient temperature related to the season of the year. According to Neryng et al. [54], greater by ca. 32% electric energy consumption per chilled product unit is recorded in the spring than in the winter. The higher temperature around the chilling chamber contributes to, e.g., slower cooling of half-carcasses after slaughter, the higher temperature in the chamber before the chilling process, and a higher value of the transfer heat.

The results of analyses of the energy consumption of production per carcasses are in contrast to those of the total amount of heat calculated (Table 5, item 9). With an almost 9-fold increase in the number of carcasses, it was necessary to remove approx. 3 times less heat units from the fast chilling chamber (39.15–15.06 kJ/carcass) and more than 5 times less heat units from the chilling tunnel (60.05–11.68 kJ/carcass). This was due to the large proportion of heat associated with the housing losses (light transmission, motor operation, door opening) in the total amount of heat, that needed to be removed from the chambers. This, in turn, was related to the time of chambers’ work. With an increase in the number of cattle/weight of carcasses, the values of the specific electricity consumption (SEC) and energy efficiency of the process (EE) calculated for the rapid chilling of stimulated meat (ES) decreased 3 times (SEC_1_ = 46.08–15.32 kJ/carcass, SEC_2_ = 0.154–0.056 kJ/kg) and increased (EE_1_ = 0.022–0.065 carcass/kJ, EE_2_ = 6.478–18.003 kg / kJ), respectively. These results indicate that with the maximum loading of the chilling chambers, the combination of innovative solutions (fast chilling + ES) is energetically and economically viable. Nunes, Silva, & Andrade [47] have demonstrated a higher Specific Energy Consumption by the meat industry in Portugal, i.e. 1208 kWh/Mg. In turn, Żywica, Banach, & Gornowicz [55] achieved Specific Energy Consumption of compressors at SEC = 19 MJ/Mg when chilling 10,320 Mg of meat with the fast two-phase method, and almost 2-fold lower SEC (SEC = 10.27 MJ/Mg) when chilling the meat with the shock method. By reducing the amount of chilled meat (6,182 Mg), they reduced electric energy consumption to 15 and 4.03 MJ/Mg, respectively. Data presented in Table 5 indicate that with the comparable weight of chilled meat, i.e. 10,553 kg and 6,616 kg, the SEC values obtained in the present study were substantially lower and reached 0.103 and 0.112 kJ/kg. This indicates that the optimal variant of beef production established in the present research is far more energy efficient than other production variants presented in literature.

Considering that one chilling cycle in a fast chilling chamber lasts 2.5h, its prolongation by 1h with carcass number of 10, by 2–3.5h with carcass numbers of 22 and 26, and by 6.5h with carcass number of 114 was due to the stable time of cooling the chambers before they were filled with half-carcasses (regardless of the amount of chilled material) and the stable chilling time of one half-carcass in the chamber. Besides, the temperature decrease by ca. 20°C in the fast chilling chamber and by ca. 18°C in the tunnel (14 to 18h, Table 1) influenced the total unitary amount of heat that had to be removed from both types of chambers (FChCh and ChT). During chilling, the total heat capacity per carcass was 99.20 kJ with 10 carcasses, and ca. 4-fold lower (26.74 kJ) with 114 carcasses (Table 5).

An indispensable side effect of the meat chilling process are its weight losses. They generate economic losses, the magnitude of which depends on the chilling method type. According to Brown et al. [56], the cost incurred by small UK plants due to meat weight losses was higher than the cost of electricity. Therefore, quick chilling methods are recommended [13] to reduce meat weight losses and achieve the best efficiency of the chilling process (shortening the time, ensuring meat safety and quality).

Results of determinations of weight losses of meat (WLM) caused by water evaporation from meat surface during its chilling with the fast two-phase method demonstrate large differences between particular days of measurements assuming various numbers of animal carcasses to be chilled (Table 1). The highest WLM (4.8%) was obtained during chilling 10 beef carcasses, presumably due to the highest volume of water evaporated from half-carcasses in the fast chilling chamber. On days 2, 3, and 4 of measurements, when the number of chilled carcasses was 21–42, the WLM ranged from 3.1 to 3.5%. The results of measurements allow concluding that smaller WLM were recorded (2.3–2.6%) with carcasses having a higher meat content, more densely packed, and touching one another in the chamber than with the lower number of chilled carcasses (Table 5). According to Klettner [57], the weight loss of meat caused by its chilling with the fast one-phase method was at 1.6% and was higher by ca. 0.3% from the WLM recorded during the shock chilling process (from –3 to –5°C/2h, 0°C/16–22h). However, the carcasses intended for chilling should not be moist because of the possibility of meat surface freezing during chilling with this method. As reported by Żywica, Banach, & Gornowicz [55], WLM obtained using the shock and fast chilling methods reached 2.2 and 3.6%, respectively.

Summing up, the analysis of energy consumption of the examined meat production variant–coupled use of HVES and fast chilling method, showed that it enabled achieving a lower value of the specific energy consumption and WLM of 2.8% at highly diversified amounts of meat chilled in the chambers, compared to the values reported by other authors. It may thus be concluded that this production variant contributes to the efficient energy consumption in the production process and sustainable company management through, among others, reducing the carbon footprint of beef and energy costs.

## Conclusions

1. The use of HVES and the fast chilling method at the slaughter line enables producing high-quality meat, reducing expenditures related to electric energy consumption, and increasing work effectiveness of cooling appliances compared to the slow and accelerated chilling methods. The coupling of these techniques is recommended for both investment and modernization undertakings in pursuit of sustainable meat production.
2. The intensive chilling of beef to ca. 20°C within 2.5h and the break in the chilling process until temperature equalization between its outer and inner layers allow the producers for getting the optimal rate of pH changes, rational quality management and changes in production organization, mainly through increasing chilling speed and reducing storage surface and expenditures for staff wages.
3. The heat balance of fast chilling chambers (FChCh+ChT) demonstrated that achieving the desired final temperature resulted in several times lower value of the specific energy consumption at the lowest amount of chilled meat, and in higher energy efficiency of the process at the highest amount of chilled meat.
4. Evaluation of the industrial quality indicators of beef and energy consumption of its production process in the variant with the coupled use of own-construction device for HVES at the slaughter stage and meat chilling with the fast method demonstrated that it allowed accomplishing the principles of sustainable beef production. In addition, it was found to indirectly contribute to mitigating the adverse effects of the meat production plant on the natural environment and to improving its image.

## Supporting information

S1 Dataset (XLS)
